# Nonmuscle Myosin IIA Regulates Intestinal Epithelial Barrier *in vivo* and Plays a Protective Role During Experimental Colitis

**DOI:** 10.1038/srep24161

**Published:** 2016-04-11

**Authors:** Nayden G. Naydenov, Alex Feygin, Dongdong Wang, John F. Kuemmerle, Gianni Harris, Mary Anne Conti, Robert S. Adelstein, Andrei I. Ivanov

**Affiliations:** 1 Department of Human and Molecular Genetics, Virginia Commonwealth University, Richmond, VA23298; 2 Department of Internal Medicine, Virginia Commonwealth University, Richmond, VA 23298; 3 Department of Medicine, University of Rochester School of Medicine, Rochester, NY; 4 Laboratory of Molecular Cardiology, NHLBI, National Institutes of Health, Bethesda, Maryland 20892; 5 VCU Massey Cancer Center, Virginia Commonwealth University, Richmond, VA 23298; 6 Virginia Institute of Molecular Medicine, Richmond, VA 23298.

## Abstract

The actin cytoskeleton is a critical regulator of intestinal mucosal barrier permeability, and the integrity of epithelial adherens junctions (AJ) and tight junctions (TJ). Non muscle myosin II (NM II) is a key cytoskeletal motor that controls actin filament architecture and dynamics. While NM II has been implicated in the regulation of epithelial junctions *in vitro*, little is known about its roles in the intestinal mucosa *in vivo*. In this study, we generated a mouse model with an intestinal epithelial-specific knockout of NM IIA heavy chain (NM IIA cKO) and examined the structure and function of normal gut barrier, and the development of experimental colitis in these animals. Unchallenged NM IIA cKO mice showed increased intestinal permeability and altered expression/localization of several AJ/TJ proteins. They did not develop spontaneous colitis, but demonstrated signs of a low-scale mucosal inflammation manifested by prolapses, lymphoid aggregates, increased cytokine expression, and neutrophil infiltration in the gut. NM IIA cKO animals were characterized by a more severe disruption of the gut barrier and exaggerated mucosal injury during experimentally-induced colitis. Our study provides the first evidence that NM IIA plays important roles in establishing normal intestinal barrier, and protection from mucosal inflammation *in vivo*.

Establishment of the intestinal epithelial barrier is a fundamental feature of healthy gut, protecting the body from the harmful contents of the gut lumen, and allowing for the regulated bidirectional transport of fluids, nutrients, and waste. Disruption of the gut barrier plays an important role in the pathogenesis of different immune disorders, such as inflammatory bowel disease (IBD), celiac disease, sepsis, and diabetes^1^,^2^. The permeability of the intestinal epithelial barrier is regulated by specialized adhesive structures known as tight junctions (TJ) and adherens junctions (AJ). AJ are responsible for initiating cell-cell contacts^3^,^4^,^5^, whereas TJ create the seal between adjacent epithelial cells^2^,^6^,^7^,^8^. These junctional complexes are composed of adhesive transmembrane proteins and, associated with them, cytosolic plaque proteins. The transmembrane proteins of epithelial TJ include members of the claudin family, occludin, and junctional adhesion molecule-A (JAM-A)^2^,^6^,^7^,^8^, whereas E-cadherin and nectins comprise the major adhesive components of AJ^3^,^4^,^5^. Cytosolic plaques of AJ and TJ contain a variety of scaffolding, signaling, and trafficking proteins including α-catenin, β-catenin, p120-catenin, and members of the *zonula occludens* (ZO) protein family^2^,^3^,^4^,^6^,^9^,^10^.

Both AJ and TJ are physically associated with the prominent cortical actin cytoskeleton^11^,^12^. This association is critical for the assembly of epithelial junctions and the establishment of a paracellular barrier. Furthermore, remodeling of the actin cytoskeleton provides the force that drives TJ and AJ disassembly induced by environmental stressors and proinflammatory agents^11^,^12^,^13^. The integrity of the perijunctional actin cytoskeleton is regulated by a large number of motor, actin binding, and signaling proteins. The roles of these cytoskeletal regulators in the function of the normal intestinal epithelial barrier, as well as in barrier dysfunctions in inflamed gut, remain poorly understood.

Perijunctional actin filaments are enriched in non muscle myosin II (NM II), a motor protein that converts the chemical energy of ATP hydrolysis into mechanical forces, thus mediating cytoskeletal tension and contractility. This protein works as a molecular ensemble consisting of two heavy chains, two essential, and two regulatory myosin light chains (RMLC)^14^,^15^. NM II heavy chains comprise the major structural component of this cytoskeletal motor. Each heavy chain has a globular head, which binds to actin filaments and hydrolyzes ATP, and an extended tail that coils together with another heavy chain tail to form rigid rod-like myosin filaments^14^,^15^. Such high-order organization of NM II is critical for the cross-linking and movement of actin filaments. Phosphorylation of RMLC by myosin light chain kinase (MLCK) or Rho kinase (ROCK) is known to alter the conformation of heavy chains, thereby increasing NM II activity^14^,^15^. A number of previous studies implicated NM II heavy chain activity and RMLC phosphorylation in controlling all the steps of junctional dynamics (assembly, maintenance, and disassembly) in cultured intestinal epithelial cell monolayers *in vitro*^16^,^17^,^18^,^19^,^20^,^21^,^22^,^23^. However, little is known regarding how NM II motor regulates the gut barrier *in vivo*. A well-established concept in the field postulates that NM II activation triggered by MLCK-dependent phosphorylation of RMLC, disrupts the epithelial barrier during intestinal inflammation^1^,^2^,^10^,^24^. This concept is based on studies demonstrating that either genetic or pharmacological alterations to MLCK activity and RMLC phosphorylation affect the integrity of the intestinal barrier and the severity of experimental colitis in some^17^,^25^,^26^, but not all animal models^25^,^27^. However, these results should be considered with caution. Despite the common belief that RMLC uniquely associates with NM II, previous reports illustrate promiscuous binding of RMLC to other proteins that include the heavy chains of unconventional myosins from classes 14,15, 18, and 19^28^, as well as non-myosin targets, such as a bile acid transporter^29^, calponin^30^, etc. Moreover, evidence exist that MLCK can modulate junctional permeability in inflamed tissue via NM II-independent mechanisms involving integrin signaling^31^.

These examples emphasize the need for more specific experimental approaches to examine the effects of NM II activity on the integrity of epithelial barriers *in vivo*. The best approach would involve targeting NM II heavy chains considering their direct and indispensable roles in actin filament assembly and dynamics. It is noteworthy that regulation of NM II activity is not limited to RMLC phosphorylation, but also involves posttranslational modifications of the heavy chains as well as their interactions with different scaffolding proteins^14^,^15^,^32^. Epithelial cells express three different NM II heavy chain isoforms, A, B, and C, each with distinct enzymatic properties and cellular functions^14^,^15^,^32^,^33^. Our previous studies have demonstrated that NM IIA plays unique roles in regulating the paracellular barrier, and AJ/TJ remodeling in intestinal and pancreatic epithelial cell monolayers *in vitro*^18^,^34^. Furthermore, mislocalization of epithelial NM IIA was observed in the inflamed colonic mucosa of Crohn’s disease patients^35^. However, the functions of NM II heavy chains (motors) in regulating epithelial barrier in healthy gut, and during mucosal inflammation, remain unexplored. This study demonstrates, for the first time, that intestinal epithelial NM IIA controls the integrity of mucosal barrier in healthy gut *in vivo,* and limits the development of experimental colitis.

## Results

### Characterization of conditional knockout of NM IIA in the intestinal epithelium

**Figure 1:**
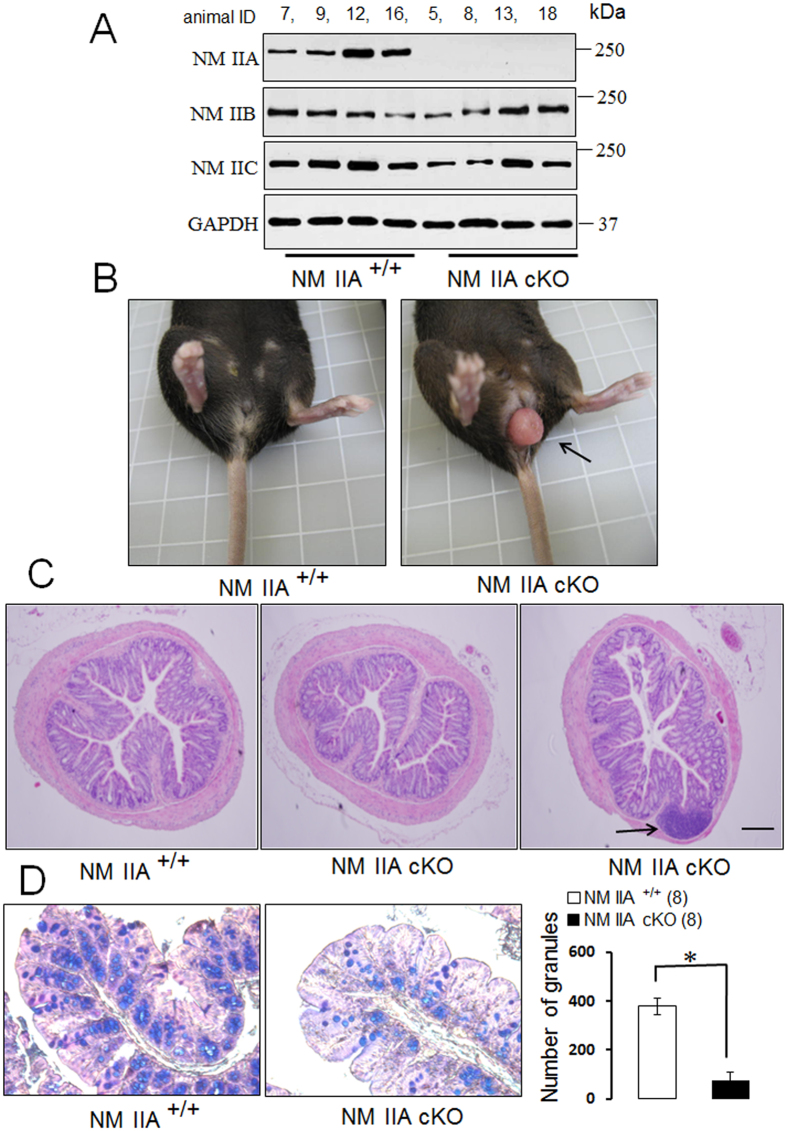
Characterization of intestinal epithelial specific NM IIA knockout mice. (**A**) Immunoblotting analysis of the expression of different NM II isoforms in colonic epithelial scrapes obtained from NM IIA^+/+^ and NM IIA cKO mice. (**B**) Spontaneous development of rectal prolapse in NM IIA cKO animals (arrow). (**C**) Normal architecture of colonic epithelium and the formation of large lymphoid aggregates (arrow) in the distal colon of NM IIA cKO mice. (**D**) Periodic acid-Shiff-Alcian Blue staining of Goblet cells in the colonic mucosa of control and NM IIA cKO animals. Numbers in parentheses indicate the number of animals in each experimental group. Data is presented as mean ± SE; *P < 0.01. Scale bar, 50 μm.

Total knockout of NM IIA in mice is embryonically lethal^36^. In order to investigate the functions of this motor protein in the gut, we generated mice with intestinal epithelium-specific knockout of NM IIA by crossing NM IIA floxed animals with villin-Cre mice. The efficiency and specificity of NM IIA knockout was examined by immunoblotting analysis of intestinal epithelial cell scrapes and tissue samples collected from different organs. Intestinal scraping is a simple and convenient technique to collect tissue fractions enriched in epithelial cell markers and depleted of mesenchymal/smooth muscle cell markers (Supplementary Figure 1A). Immunoblotting analysis confirmed the selective loss of NM IIA expression in colonic and ileal epithelium without significant changes to its expression in the brain, kidney, lungs, and liver (Fig. 1A, Supplementary Figure 1). This knockout was specific for NM IIA and did not affect the expression of closely-related NM IIB and NM IIC isoforms (Fig. 1A). NM IIA flox/villin Cre homozygous animals (abbreviated hereafter as NM IIA cKO) appeared to be healthy. They gained body weight similar to control littermates and did not show spontaneous diarrhea or rectal bleeding (data not shown). The only phenotypic abnormality of NM IIA cKO mice was the development of rectal prolapses that were observed in approximately 52% of NM II cKO mice, but not in NM IIA^+/+^ or heterozygous animals (Fig. 1B, Table 1). Similar rectal prolapses were previously reported in different murine models of colitis, including interleukine-10 knockout mice and mice with the Th1 mucosal immune response to trinitrobenzoic acid^37^,^38^,^39^,^40^. The development of rectal prolapses is considered a sign of mucosal inflammation, although this phenotype is not an obligate consequence of inflammation, and was observed only in a fraction (8–67%) of the animals with colitis^37^,^38^,^39^,^40^.

Histological analysis reveals that intestinal epithelium-specific loss of NM IIA did not induce gross morphological alterations of normal colonic mucosa (Fig. 1C, middle image). However, approximately 42% of NM IIA cKO mice developed large lymphoid aggregates in the distal colon (Fig. 1C, arrow, and Table 1). Such lymphoid aggregates, along with the described rectal prolapses, could be indicative of low-scale mucosal inflammation in NM IIA cKO animals^40^,^41^. Another effect of intestinal epithelial NM IIA knockout was a dramatic decrease in the number of mucin granules in colonic Goblet cells, as indicated by Periodic Acid-Shiff and Alcian Blue staining (Fig. 1D). Such depletion of mucin granules was not accompanied by decreased mRNA expression of mucin-2 or molecular components of the Notch signaling pathway (Notch1, Hes1, and Math1), which plays a key role in Goblet cell differentiation (Supplementary Figure 2A). Therefore, the loss of mucin granules in NM IIA cKO colon is unlikely to reflect abnormal Goblet cell differentiation, but rather could be caused by either defective granule biogenesis or accelerated granule release due to mild mucosal inflammation. Moreover, the expression of Paneth cell markers, lysozyme and defensins, and the morphology of Paneth cell granules were not affected by the conditional loss of NM IIA in small intestine (Supplementary Figure 2B,C).

### Intestinal specific knockout of NM IIA disrupted normal gut barrier *in vivo*

**Figure 2:**
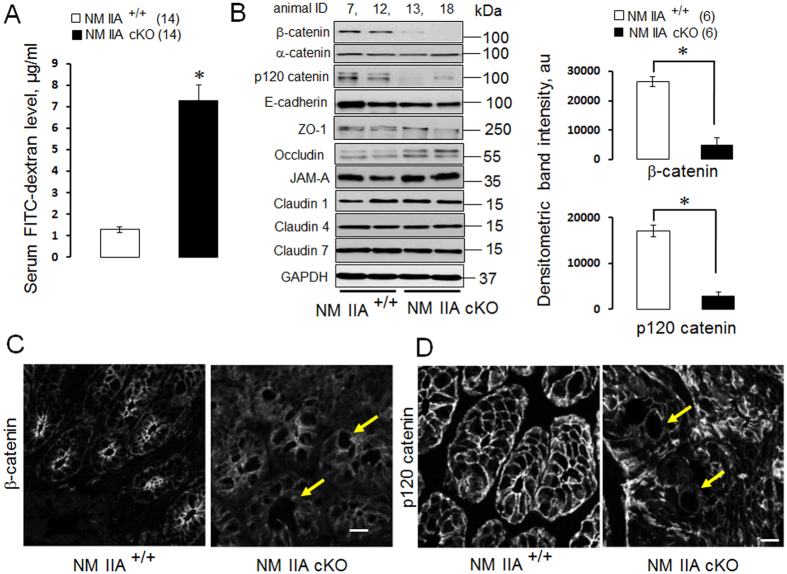
The effects of intestinal epithelial specific deletion of NM IIA on the permeability of normal mucosal barrier and the structure of epithelial junctions. (**A**) The intestinal permeability of unchallenged NM IIA^+/+^ and NM IIA cKO mice was examined by measuring the trans-mucosal flux of FITC-dextran. (**B**) Immunoblotting analysis and selective densitometric quantification of AJ and TJ protein expression in the colonic epithelial scrapes of NM IIA^+/+^ and NM II cKO animals. (**C,D**) Immunofluorescence labeling and confocal microscopy of β-catenin (**C**) and p120 catenin (**D**) in colonic sections. Arrows show decreased labeling intensity of AJ proteins in the colonic crypts of NM IIA cKO mice. Data is presented as mean ± SE; *P < 0.01. Scale bar, 10 μm.

**Figure 3:**
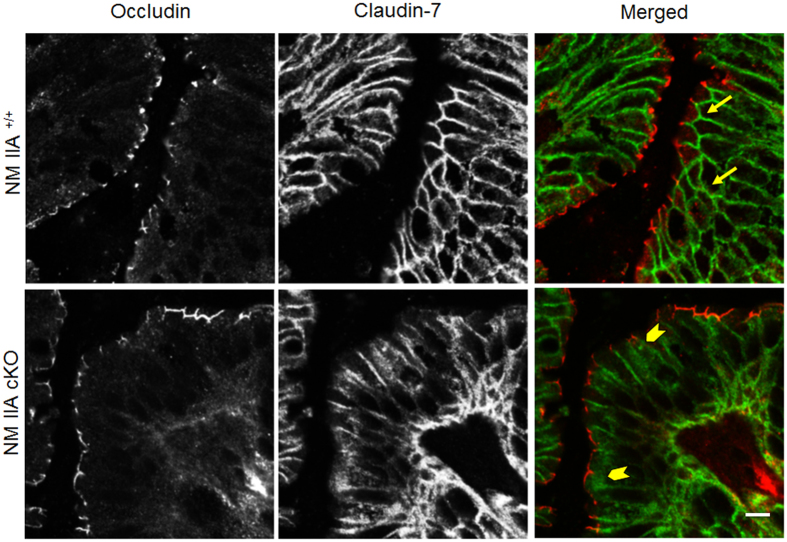
The loss of intestinal epithelial NM IIA triggers mislocalization of claudin-7 in colonic mucosa. Colonic sections of control and NM IIA cKO animals were dual-immunolabeled for occludin (red) and claudin-7 (green), and examined by confocal microscopy. Arrows indicate normal labeling of claudin-7 at the intercellular junctions of control mice. Arrowheads point out the cytoplasmic translocation of claudin-7 in the colonic epithelium of NM IIA cKO mice. Scale bar, 10 μm.

**Figure 4:**
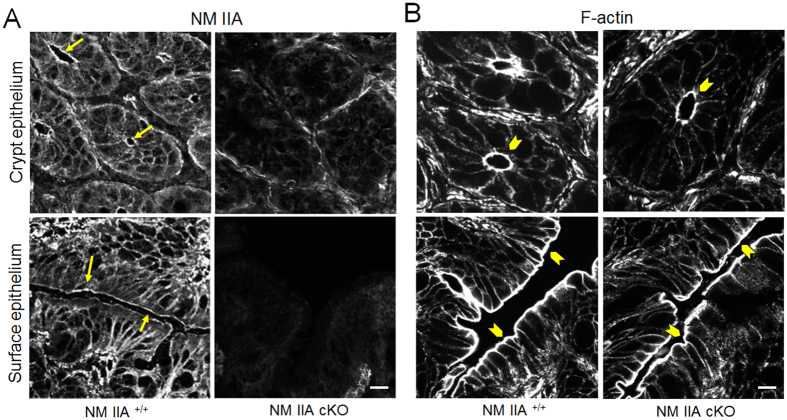
Intestinal specific deletion of NM IIA does not affect the organization of the actin cytoskeleton. Immunofluorescence and fluorescence labeling of NM IIA and F-actin, respectively, in different areas of the colonic epithelium of control and NM IIA cKO mice. Arrows indicate localization of NM IIA in NM IIA^+/+^ epithelium, whereas arrowheads point to intact F-actin bundles in NM IIA^+/+^ and NM IIA-depleted colonic mucosa. Scale bar, 10 μm.

Considering the previous reports that inhibition of the NM II motor induces permeability defects in model intestinal epithelial monolayers^18^,^20^ we sought to investigate the effects of NM IIA knockdown on the integrity of gut barrier *in vivo*. Remarkably, unchallenged NM IIA cKO mice demonstrated approximately 6-fold increase in the transmucosal flux of FITC-dextran as compared to NM IIA^+/+^ animals, thereby indicating a leaky gut barrier (Fig. 2A). Such barrier disruption was accompanied by a selective decrease in the expression of two AJ cytoplasmic plaque proteins, β-catenin and p120 catenin (Fig. 2B), whereas expression of E-cadherin and all other major TJ proteins remained unchanged. Immunofluorescence labeling revealed a loss in apical junctional staining of β-catenin and p120 catenin in colonic crypts of NM IIA cKO mice (Fig. 2C,D arrows). Furthermore, a marked redistribution of claudin-7 from the intercellular junctions into cytosolic compartments, was observed in the colonic mucosa of NM IIA cKO animals (Fig. 3, arrowheads). In contrast, junctional localization of E-cadherin, occludin, and ZO-1, was not changed (Fig. 3; Supplementary Figure 3). Immunofluorescence labeling demonstrated enrichment of NM IIA at the apical and lateral plasma membrane in the colonic epithelium of NM IIA^+/+^ mice (Fig. 4A arrows), and loss of this labeling in NM IIA cKO animals. Interestingly, the loss of NM IIA did not affect the assembly of perijunctional F-actin bundles in either colonic crypt or surface epithelium (Fig. 4B arrowheads). Immunofluorescence analysis of the localization of other NM II isoforms revealed enrichment of NM IIC at the apical pole of NM IIA^+/+^ enterocytes, while NM IIB labeling was limited to the basal epithelial surface and *lamina propria* cells (Supplementary Figure 4). This localization of NM IIB and NM IIC was not altered in the colonic sections of NM IIA cKO mice (Supplementary Figure 4).

### Intestinal epithelial specific knockout of NM IIA increased the severity of experimental colitis

**Figure 5:**
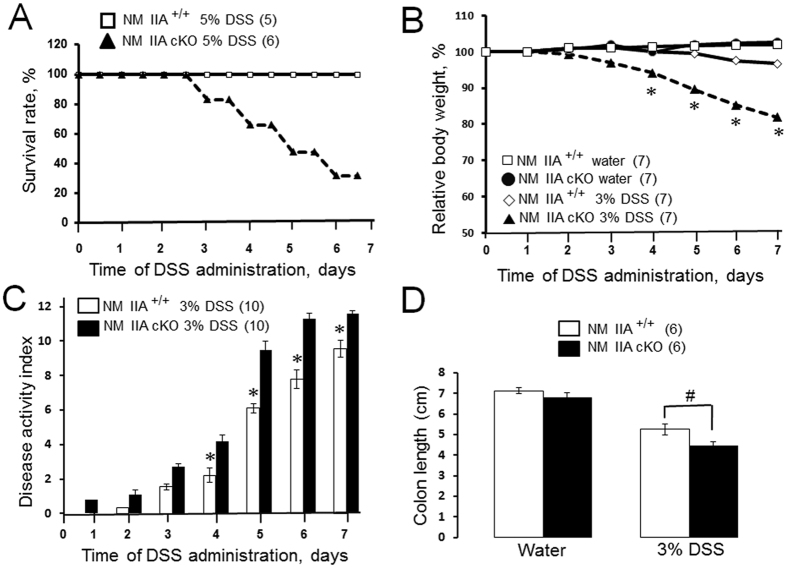
Intestinal epithelial-specific knockout of NM IIA exacerbates disease symptoms during experimental colitis. **(A)** NM IIA^+/+^ and NM IIA cKO mice were exposed to 5% DSS in drinking water for 7 days. A Kaplan-Meyer plot shows colitis-induced animal mortality. NM IIA^+/+^ and NM IIA cKO mice were exposed to 3% DSS in drinking water, or water alone, for 7 days. **(B)** Body weight, and **(C)** disease activity index were recorded daily. **(D)** Colon length was measured at the end of the experiment. Data is presented as mean ± SE. Number of animals in each experimental group is shown in parentheses. *P < 0.01, ^#^P < 0.05

**Figure 6:**
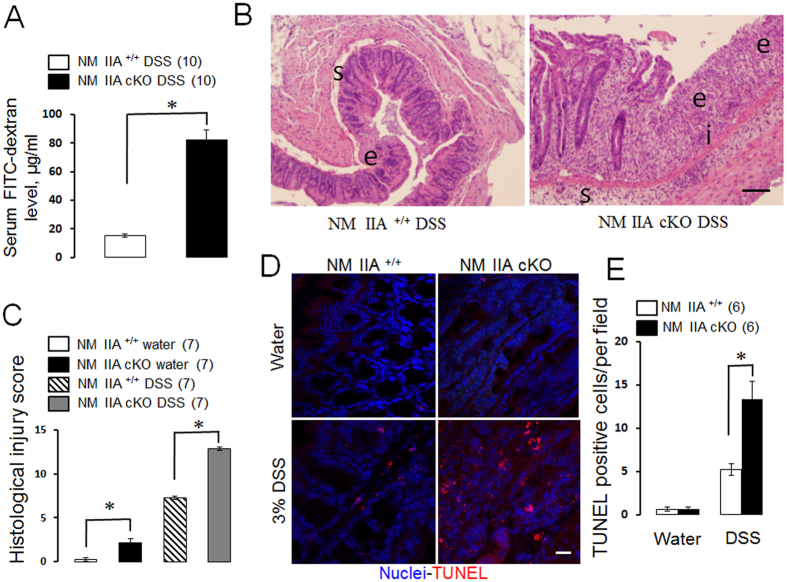
Intestinal epithelial-specific knockout of NM IIA increases tissue injury and cell apoptosis during experimental colitis. NM IIA^+/+^ and NM IIA cKO mice were exposed to 3% DSS in drinking water, or water alone, for 7 days. **(A)** Intestinal permeability was determined by measuring transmucosal flux of FITC-dextran**. (B,C)** Hematoxylin & eosin staining was used to evaluate epithelial integrity and to calculate the tissue injury index. Changes in the tissue architecture are indicated by letters: e, epithelial erosion; i, leukocyte infiltration; s, submucosal edema. Scale bar, (**B**) 50 μm. **(D,E)** Apoptotic cells were visualized using TUNEL assay (red). Nuclear counter-staining (blue) was used to visualize the position of individual cells. Data is presented as mean ± SE. Number of animals in each experimental group is shown in parentheses. *P < 0.01. Scale bar, (**D**) 10 μm.

Since disruption of the intestinal epithelial barrier plays an important role during mucosal inflammation we next sought to investigate whether loss of intestinal epithelial NM IIA affects the development of experimental colitis. Administration of a high (5%) dose of dextran sodium sulfate (DSS) induced approximately 80% mortality in NM IIA cKO mice without causing animals’ death in the control group (Fig. 5A). A lower dose of DSS (3%) triggered more severe intestinal disease in NM IIA cKO mice, as manifested by more pronounced body weight loss (Fig. 5B), a significantly higher disease activity index (Fig. 5C), and shortening of the colon (Fig. 5D). DSS administration disrupted intestinal mucosal barriers causing a marked increase in transmucosal passage of FITC-dextran (compare Figs 6A and 2A). Remarkably, DSS-induced mucosal permeability was approximately 5-fold higher in NM IIA cKO animals, as compared to their NM IIA^+/+^ littermates (Fig. 5A). Histological analysis demonstrated mucosal damage and inflammation in the colonic mucosa of DSS-treated NM IIA^+/+^ mice that included submucosal edema, crypt hyperplasia, leukocyte infiltration, and focal epithelial erosion (Fig. 6B,C; Supplementary Figure 5). This mucosal injury was much more pronounced in the tissue samples of DSS-exposed NM IIA cKO mice, as characterized by the marked loss of epithelial cells in the distal colon (Fig. 6B,C; Supplementary Figure 5). Consistent with our histology data, TUNEL assay of whole colonic segments (Fig. 6 D,E), and immunoblotting analysis of colonic epithelial scrapes (Supplementary Figure 6), revealed much more pronounced apoptosis in the colonic mucosa of DSS-treated NM II cKO mice, as compared to similarly-treated control animals. Together, this data strongly suggests that loss of intestinal epithelial NM IIA sensitizes mice to DSS-induced colitis.

### Intestinal epithelial specific knockout of NM IIA exacerbated the inflammatory response in colonic mucosa

**Figure 7:**
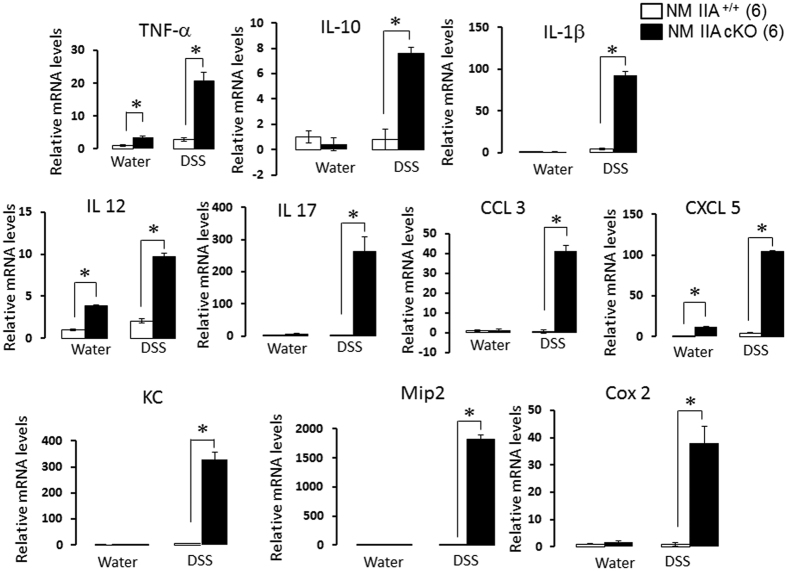
Intestinal epithelial-specific knockout of NM IIA exaggerates cytokine and chemokine expression in normal and inflamed colon. NM IIA^+/+^ and NM IIA cKO mice were exposed to either 3% DSS in drinking water, or water alone, for 4 days. Colonic samples were harvested and the expression of different cytokines, chemokines and Cox2 was determined by real-time quantitative RT-PCR. Data is presented as mean ± SE. Number of animals in each experimental group is shown in parentheses. *P < 0.01.

We next sought to investigate if the increased severity of the disease and more pronounced mucosal damage of DSS-treated NM IIA cKO animals was due to enhanced mucosal inflammation. First, we measured the expression of different proinflammatory cytokines during the early stages of colitis (4 days of DSS administration). Interestingly, quantitative RT-PCR analysis revealed a modest but significant increase in the mRNA levels of proinflammatory cytokines, tumor necrosis factor alpha (TNFα), interleukin (IL-12), and a chemokine, C-X-C motif ligand 5 (CXCL5), in the normal colonic mucosa of NM IIA cKO mice as compared to their NM IIA^+/+^ littermates (Fig. 7). This further supports our suggestion that a low-scale mucosal inflammation is present in these animals. Moreover, the mRNA expression of virtually all measured cytokines (TNFα, IL-1β, IL-10, IL-12 and IL-17), chemokines (CXCL5, C-C motif ligand 3 (CCL3); keratinocyte-derived chemokine (KC), macrophage inflammatory protein 2 (Mip2) and cyclooxygenase (Cox) 2 was dramatically upregulated in the colonic mucosa of DSS-treated NM IIA cKO mice, as compared to their DSS-exposed controls (Fig. 7).

**Figure 8:**
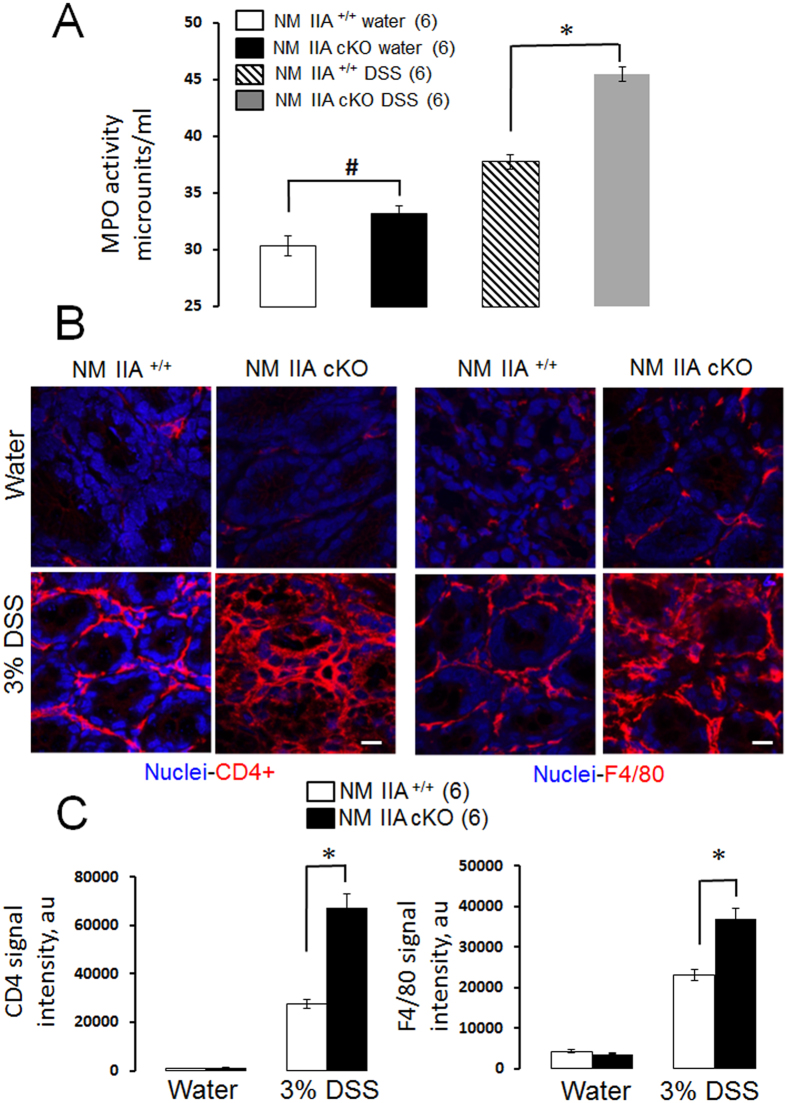
Loss of NM IIA in the intestinal epithelium increases colonic infiltration of leukocytes during experimental colitis. Colonic samples of NM IIA^+/+^ and NM IIA cKO mice were collected on day 7 of 3% DSS or water administration. (**A**) MPO activity was measured as a marker for neutrophil infiltration. (**B,C**) Macrophages and T cells were visualized by immunolabeling their specific cellular markers, F4/80, and CD4, respectively (red). Nuclear counter-staining (blue) was used to visualize the position of individual cells. Data is presented as mean ± SE. Number of animals in each experimental group is shown in parentheses. *P < 0,01, ^#^P < 0,05. Scale bar, 10 μm.

Finally, we sought to determine whether loss of NM IIA affects the recruitment of different classes of leukocytes into inflamed colonic mucosa. We utilized a myeloperoxidase (MPO) activity assay and immunolabeling of F4/80 and CD4 antigens to detect neutrophils, macrophages, and T-lymphocytes respectively. A significant increase in MPO activity was observed in the colonic tissue of unchallenged NM IIA cKO mice (Fig. 8A). Additionally, NM IIA cKO animals showed much higher induction of MPO activity after 7 days of DSS administration. Similarly, DSS colitis in NM IIA cKO mice was accompanied by a more pronounced mucosal recruitment of macrophages and CD4-positive T lymphocytes (Fig. 8B,C). Together this data provides further evidence of low-scale mucosal inflammation in unchallenged NM IIA cKO mice, and reveals the enhanced inflammatory response of these animals during DSS-induced colitis.

### Intestinal epithelial specific knockout of NM IIA enhanced the expression of transforming growth factor-beta and the accumulation of mucosal IgA

**Figure 9:**
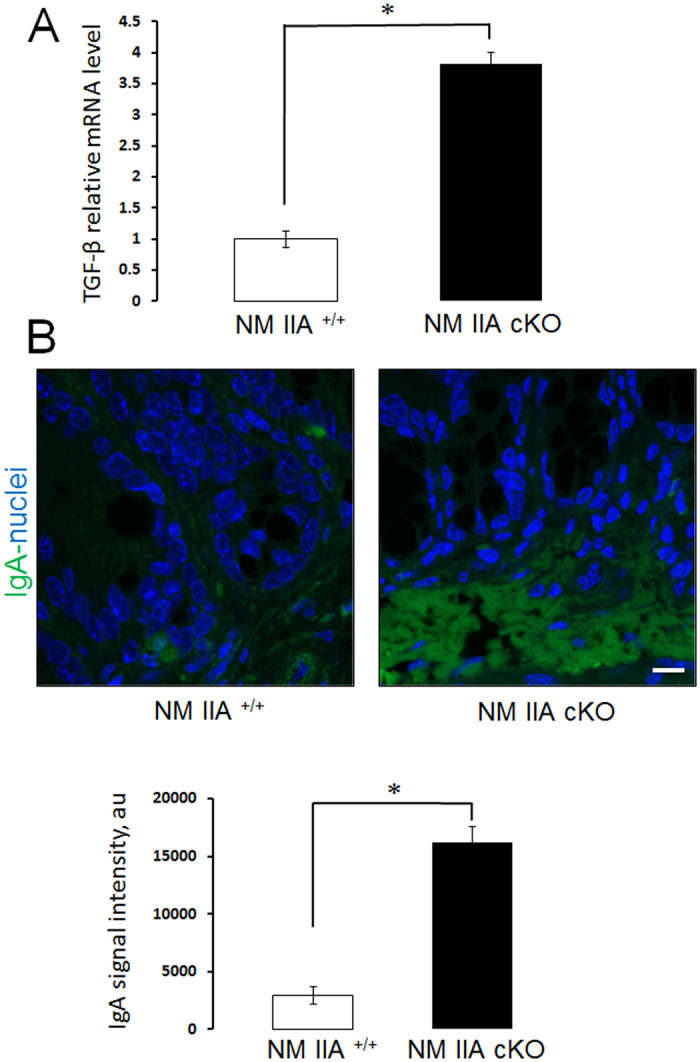
Loss of NM IIA in the intestinal epithelium results in the increase of TGF-β expression and the accumulation of IgA. **(A)** TGF-β mRNA expression in the colonic tissue of unchallenged NM IIA^+/+^ and NM IIA cKO mice was determined using real-time RT**-**PCR analysis. (**B**) IgA (green) was visualized in colonic sections by immunolabeling and confocal microscopy. Nuclear counter-staining (blue) was used to visualize the position of individual cells. Data is presented as mean ± SE (n = 5); *P < 0,01. Scale bar, 10 μm.

The persistence of low-scale mucosal inflammation, without the development of spontaneous colitis observed in NM IIA cKO mice, suggests the existence of a protective compensatory response in their intestinal mucosa. A protective immune response that involves transforming growth factor beta (TGF-β) driven production of IgA was recently described in mice with total JAM-A knockout^42^. Therefore, we sought to investigate if a similar compensatory response exists in NM IIA cKO mice. Quantitative RT-PCR analysis of colonic tissue demonstrated a more than 3 fold increase in TGF-β expression in unchallenged NM IIA cKO mice, as compared to NM IIA^+/+^ littermates (Fig. 9A). Moreover, immunofluorescence analysis revealed an accumulation of IgA in the NM IIA-depleted intestinal mucosa (Fig. 9B,C). This data suggests that upregulation of TGF-β and IgA expression may protect the animals from spontaneous colitis under ‘leaky’ intestinal epithelial barrier conditions.

## Discussion

NM II motors are essential regulators of cellular homeostasis and tissue integrity, playing multiple roles in cell polarity, division, motility, and mechanotransduction^14^,^15^. The predominant body of studies focuses on the activity and regulation of mammalian NM II in cultured cells, *in vitro*^12^,^13^,^14^,^15^,^32^. However, physiological functions of these motor proteins in different tissues remain poorly understood. The present study provides the first evidence that NM IIA acts as a positive regulator of the intestinal epithelial barrier in normal gut and suppresses intestinal mucosal inflammation *in vivo*. We successfully generated a selective knockout of NM IIA in the intestinal epithelium (Fig. 1; Supplementary Figure 1). Characterization of unchallenged NM IIA cKO animals revealed several important phenotypes that included increased mucosal permeability (Fig. 2A), decreased expression and mislocalization of some junctional proteins (Figs 2B–D and 3), loss of mucin granules in Goblet cells (Fig. 1D), and the development of low-scale mucosal inflammation (Figs 1B,C, 7 and 8A). The intestinal epithelial phenotypes of NM IIA cKO mice appear to be relatively mild and did not involve global disruption of AJ/TJ integrity or abnormal organization of the perijunctional cytoskeleton. This is consistent with our previous results obtained in NM IIA-depleted cultured intestinal epithelial cell monolayers^18^. A partial preservation of AJ and TJ structure in NM IIA-depleted intestinal epithelium most likely reflects the compensatory effects of the remaining NM II isoforms, most notably, NM IIC. Indeed, NM IIC was found to account for up to 20% of total NM II in the intestinal epithelium^33^ and it is enriched at intestinal epithelial apical junctions *in vitro*^18^ and *in vivo* (Supplementary Figure 4).

The observed decreased expression of β-catenin and p120 catenin in the colonic mucosa of NM IIA cKO mice is unusual for two reasons. First, it is not paralleled by the decreased expression or altered localization of E-cadherin (Fig. 2B; Supplementary Figure 3), thereby contrasting with the reported loss of E-cadherin in p120-depleted intestinal epithelial cells^43^ and tissue^44^. However NM IIA cKO mice do not show a complete loss of intestinal epithelial p120 catenin, and the remaining pool of this protein could be sufficient to stabilize E-cadherin. Second, the decreased expression of β-catenin and p120-catenin appears to be a specific effect of NM IIA knockout *in vivo,* since the expression of these AJ scaffolds was not changed in intestinal epithelial cells after either siRNA-mediated knockdown or CRISPR/Cas9-mediated knockout of NM IIA *in vitro*^18^ (and data not shown). One can suggest, therefore, that downregulation of β-catenin and p120 catenin could be indirect effects of NM IIA depletion that can be linked to the disruption of the gut barrier and increased exposure to luminal microbiota.

Despite having a defective intestinal epithelial barrier, NM IIA cKO mice did not develop spontaneous colitis. We believe this could be explained by the development of a protective mechanism, similar to the mechanism recently described in JAM-A knockout mice^41^,^42^. NM IIA cKO and JAM-A knockout mice showed similar physiological and biochemical abnormalities that include: (i) leaky gut barrier; (ii) lymphoid aggregates in the colonic mucosa; (iii) increased expression of colonic TGF-β; (iv) and the accumulation of intestinal mucosal IgA. A comprehensive genetic and immunological analysis of JAM-A mice concluded that they have an activated CD4 T-cell response resulting in TGF-β-dependent secretion of IgA. This limits bacterial translocation and intestinal mucosal inflammation under conditions of a compromised intestinal epithelial barrier^42^. Our data suggests that a similar compensatory mechanism may limit the inflammatory response in the intestinal mucosa of unchallenged NM IIA cKO animals (Fig. 9).

The present study reveals a key role of NM IIA in attenuating mucosal inflammation and tissue injury in DSS-challenged mice (Figs 5, 6, 7, 8; Supplementary Figures 5 and 6). This anti-inflammatory role is likely to involve the barrier-stabilizing activity of this cytoskeletal motor, since increased mucosal inflammation correlated with a more dramatic breakdown of gut barrier in NM IIA cKO animals (Fig. 6A). It is unlikely that the more severe mucosal damage and inflammation in NM IIA cKO mice reflect the increased sensitivity of epithelial cells to DSS toxicity. Indeed NM II inhibition was shown to have prosurvival effects on mammalian stem cells and intestinal enteroids^45^,^46^. Furthermore, we did not observe increased death of NM IIA knockout HT-29 cells after direct exposure to 3% DSS *in vitro* (data not shown).

Our data purporting the anti-inflammatory role of intestinal epithelial NM IIA is in variance with a recent report suggesting that inhibition of intestinal NM IIA alleviates DSS-induced colitis^46^. These authors attenuated NM IIA activity by using either a pharmacological NM II inhibitor, blebbistatin, or via monoallelic deletion of NM IIA in the intestinal epithelium (NM IIA flox/+/Villin-cre)^46^. However, the effects of blebbistatin on DSS colitis are difficult to interpret, since this inhibitor directly blocks leukocyte infiltration and activity in the intestinal mucosa. Furthermore, monoallelic NM IIA deletion in mice neither decreased expression of this protein, nor did it alter animal responsiveness to DSS in our experiments (data not shown). We believe that NM IIA cKO animals developed in our study represent the best tool to investigate the *in vivo* functions of this cytoskeletal motor in the intestinal epithelium.

The increased permeability of normal and inflamed intestinal mucosa observed in NM IIA cKO mice challenges the current dogma that only NM IIA activation drives opening of the gut barrier under physiological conditions and during intestinal inflammation. Taken together, our data and previous studies indicate that a balanced, intermediate activity of NM IIA is essential for the integrity of the normal gut barrier. Both excessive activation and inactivation of NM II motor should increase intestinal permeability. While the external stimuli and signaling events that activate intestinal epithelial NM II have been intensively investigated^1^,^9^,^47^,^48^, little is known about how this motor could be inhibited in gastrointestinal diseases. A number of different mechanisms controlling the assembly and activity of NM II motors have been described *in vitro*. These mechanisms include regulation of expression, chaperon-assisted folding, heavy chain phosphorylation, and associations with multiple myosin-binding proteins^15^,^32^. Further study is required to elucidate how these mechanisms control NM IIA functions in the gut under physiological conditions and during gastrointestinal diseases.

## Materials and Methods

### Antibodies and other reagents

The following primary monoclonal (mAb) and polyclonal (pAb) antibodies were used to detect cytoskeletal, junctional, and leukocyte proteins: anti-p120-catenin and E-cadherin, Ly-6 and CD4 mAbs (BD Biosciences, San Jose, CA); anti-occludin, JAM-A, ZO-1, Claudin-1 and 7 pAbs, and Claudin-4 mAb (Life Technologies, Waltham, MA); anti-total actin (clone C4) mAb (EMD Millipore, Billerica, MA); anti-GAPDH (14C10) mAb and active caspase 3 pAb (Cell Signaling, Beverly, MA); anti-β-catenin pAb (Sigma-Aldrich, Saint Lois, MO); anti-α-catenin mAb (Abcam, Cambridge, MA); anti-NM IIA, NM IIB and NM IIC pAbs (Covance, Princeton, NJ); F4/80 mAb (Bio-Rad Laboratories, Hercules, CA); goat anti-E-cadherin pAb (R&D Systems, Minneapolis, MN); anti- lysozyme pAb (Santa Cruz, Dallas, TX); FITC conjugated rat anti-mouse IgA (eBioscience, San Diego, CA). Alexa Fluor-488-conjugated donkey anti-rabbit and donkey anti-goat, Alexa Fluor-555-conjugated donkey anti-mouse and goat anti-rat secondary antibodies, and Alexa Fluor-488 and 555-labeled phalloidin were obtained from Life Technologies. Horseradish peroxidase-conjugated goat anti-rabbit and anti-mouse secondary antibodies were acquired from Bio-Rad Laboratories. All other chemicals were obtained from Sigma-Aldrich.

### Animals

In order to establish a conditional knockout of NM IIA in the intestinal epithelium, NM II A^flox/flox^ mice on a C57BL/6/129/Sv background^49^ were crossed with villin-Cre animals (Jackson Laboratory, stock #004586). In these villin-Cre mice, a 12.4 kb fragment of mouse villin 1 promoter directs transgenic recombination in both the small intestine and the colon^50^. The animal colony was maintained under pathogen-free conditions in the vivarium of VCU Medical Center. Standard feed and tap water were available, *ad libitum*. The mouse room was on a 12 h light/dark cycle (lights on at 7:00 A.M.). At the beginning of experiments, mice weighed 18–25 g, and there was no meaningful difference between the body masses of mice of different genotypes. All procedures were conducted under an animal research protocol (AD10000458) approved by the Virginia Commonwealth University Animal Care and Use Committee in accordance with the National Institutes of Health Animal Care and Use Guidelines.

### Induction and characterization of dextran sulfate sodium (DSS) colitis

Experimental colitis was induced in 8–10 week old NM II A^flox/flox^/villin Cre+ mice (abbreviated as NM IIA cKO) by administering either a 5% or 3% (w/v) solution of DSS (molecular weight 40 kDa; MP Biomedicals, Santa Ana, CA) in drinking water, *ad libitum*. Either NM IIA^flox/flox^ or villin-Cre only littermates were used as controls (referred as NM IIA^+/+^). Unchallenged animals received tap water. Both male and female mice were used at roughly equal numbers in this study. The animals were weighed daily and monitored for signs of intestinal inflammation. The disease activity index was calculated as previously described, by averaging numerical scores of body weight loss, stool consistency, and intestinal bleeding^51^. With regards to body weight, no weight loss was scored as 0, loss of 1–5% was scored as 1, 5–10% weight loss as 2, 10–15% as 3, and more than 15% weight loss was scored as 4. For stool consistency, well-formed pellet was scored as 0, soft and semi-formed stool as 2, and liquid stool or diarrhea scored as 4. For intestinal bleeding, no blood was scored as 0, hemoccult-positive stool as 2, and gross rectal bleeding was scored as 4. On day 7 of DSS administration, animals were euthanized and their colonic tissue was separated into several segments. The samples were either fixed in 4% paraformaldehyde (PFA), snap frozen in liquid nitrogen, or embedded in OCT and frozen for subsequent histological and biochemical examination. PFA-fixed samples were paraffin embedded, sectioned, and stained with hematoxylin and eosin (H&E). The tissue injury index was calculated based on microscopic examination of H&E sections, as previously described^52^. The index represents the sum of individual scores reflecting leukocyte infiltration, crypt hyperplasia/inflammation, and epithelial erosion.

### Measurement of epithelial barrier permeability *in vivo*

*In vivo* permeability assay was performed in NM II A cKO and NM IIA^+/+^ animals receiving either 3% DSS for 7 days or water. Animals were gavaged with 4,000 Da FITC-labeled dextran (60 mg/100 g body weight) and euthanized 3 h later for blood collection via cardiac puncture. Blood serum was obtained by centrifugation, and FITC fluorescence intensity was measured using a Victor3 V plate reader (Perkin Elmer, Waltham, MA) with excitation and emission wavelengths at 485 and 544 nm, respectively. The value of FITC-dextran-free serum was subtracted from each measurement. The concentration of FITC-dextran in blood serum was calculated using SigmaPlot v12.5 software, based on a plotted standard curve prepared via serial dilutions of the stock solution of FITC-dextran in phosphate buffered saline (PBS).

### Immunoblotting analysis

Mice were euthanized, colonic and ileal segments were harvested, longitudinally opened, and washed with ice-cold PBS. Intestinal epithelium was collected by scraping the exposed interior with glass slides, then snap frozen in liquid nitrogen for further analysis. Intestinal epithelial scrapes were lysed and homogenized in RIPA buffer containing a protease inhibitor cocktail and phosphatase inhibitor cocktails 2 and 3 (Sigma-Aldrich). Samples were diluted with 2x SDS sample loading buffer and boiled. SDS-polyacrylamide gel electrophoresis was conducted using standard protocols with an equal amount of total protein loaded per lane (10 or 20 μg), followed by immunoblotting on nitrocellulose membrane. Protein expression was quantified via densitometry using Image J software (National Institutes of Health, Bethesda MD).

### Quantitative real-time RT-PCR

Total RNA was isolated from the whole colonic segments of NM IIA^+/+^ and NM IIA cKO animals using an RNeasy mini kit (QIAGEN, Valencia, CA) followed by DNase treatment to remove genomic DNA. Total RNA (1 μg) was reverse transcribed using an iScript cDNA synthesis kit (Bio-Rad Laboratories). Quantitative real-time RT-PCR was performed using iTaq Universal SYBR Green Supermix (Bio-Rad Laboratories) and a 7900HT Fast Real-time PCR System (Applied Biosystems, Foster City, CA). The primer sequences are listed in the Supplemental Table 1. The threshold cycle number (Ct) for specific genes of interest and a housekeeping gene were determined based on the amplification curve representing a plot of the fluorescent signal intensity versus the cycle number. Relative expression of each gene was calculated by a comparative Ct method that is based on the inverse proportionality between Ct and the initial template concentration (2^−ΔΔCt^), as previously described^53^. This method is based on two-step calculations of ΔCt = Ct_target gene_ − Ct_GAPDH_ and ΔΔCt = ΔCt_e_ − ΔCt_c_, where index e refers to the sample from any DSS or water-treated NM II A cKO, or NM IIA^+/+^ mice, and index c refers to the sample from a water-treated NM IIA^+/+^ animal assigned as an internal control.

### Immunofluorescence labeling, TUNEL assay, confocal microscopy and histochemistry

Colonic frozen sections were fixed with 95% ethanol to visualize junctional proteins, NM II isoforms, and leukocyte markers. PFA fixed and paraffin embedded sections were used for F-actin and IgA labeling. Following standard deparaffinization and antigen retrieval, sections were blocked for 60 minutes in Hanks HEPES-buffered salt solution containing 1% bovine serum albumin, followed by a 60 min incubation with primary antibodies. Samples were then washed and incubated with Alexa dye-conjugated secondary antibodies for 60 minutes, rinsed with blocking buffer, and mounted on slides with ProLong Antifade mounting reagent with or without DAPI (Life Technologies). F-actin was visualized by 60 min labeling with Alexa-555-labeled phalloidin. TUNEL assay was performed using an ApopTag Fluorescein *In Situ* Apoptosis Detection Kit (EMD Millipore), according to the manufacturer’s instructions. Labeled cell monolayers and tissue sections were imaged using a Zeiss LSM 700 Laser Scanning Confocal Microscope (Carl Zeiss Microscopy LCC, Peabody, MA). The Alexa Fluor 488 and 555 signals were acquired sequentially, in frame-interlace mode, to eliminate cross talk between channels. Images were processed using Zen 2011 software and Adobe Photoshop. To quantify the results of tissue TUNEL assay, T-cell marker (CD4) and macrophage marker (F4/80) labeling, signal intensity was measured on the mucosal surface and crypt areas of each animal’s sample. Mean values were calculated by averaging signal intensities obtained from the tissue samples of 5–7 different animals from each experimental group. The animal numbers are presented in the figures. Histochemical visualization of Goblet cells was performed using a combined Periodic Acid-Shiff and Alcian Blue staining kit (Sigma-Aldrich), according to the manufacturer’s instructions. Stained tissue sections were examined using a light microscope (Olympus BX41, Japan). Mucin granules were counted manually in blind fashion and, data are presented as number of granules per microscopic field.

### Myeloperoxidase (MPO) activity assay

MPO activity in the colons of unchallenged and DSS colitis induced animals was determined fluorometrically using a kit from Sigma-Aldrich, as described by the manufacturer. Briefly, frozen colonic samples were homogenized in MPO assay buffer. After brief centrifugation, supernatants were assayed for MPO activity using a fluorigenic substrate, aminophenyl fluorescein, and analysis on the Victor3 V plate reader with excitation and emission wavelengths 485 and 544 nm, respectively. A standard curve was created using serial dilutions of known concentrations of the kit enzyme. One unit of MPO activity was determined as the amount of the enzyme that oxidizes the MPO substrate to yield 1.0 μmole of fluorescein per minute at room temperature.

### Statistical analysis

Data are given as mean ± SEM. The statistical significance of the difference between 2 sets of data was evaluated by the two tailed unpaired Student’s T-test. Differences among three or more groups were tested for statistical significance using one way ANOVA (SigmaPlot 12.5 package). Statistical significance was accepted at P < 0.05.

## Additional Information

**How to cite this article**: Naydenov, N. G. *et al*. Nonmuscle Myosin IIA Regulates Intestinal Epithelial Barrier *in vivo* and Plays a Protective Role During Experimental Colitis. *Sci. Rep.* **6**, 24161; doi: 10.1038/srep24161 (2016).

## Supplementary Material

Supplementary Information

## Figures and Tables

**Table 1:** Incidence of spontaneous rectal prolapse and lymphoid aggregates in control and NM IIA cKO mice.

**Phenotype**	**NM IIA**^**+/+**^ **(%)**	**NM IIA cKO (%)**
Prolapse	0/35 (0)	20/38 (52)
Lymphoid aggregates	1/17 (6)	8/19 (42)
