# Supplementary material for: Nonmuscle Myosin IIA Regulates Intestinal Epithelial Barrier in vivo and Plays a Protective Role During Experimental Colitis
Source: Sci Rep. 2016 Apr 11;6:24161. doi: 10.1038/srep24161 (PMC4827066; doi:10.1038/srep24161)
Supplement: Supplementary Information [file srep24161-s1.pdf]

SUPPLEMENTARY INFORMATION

NONMUSCLE MYOSIN IIA REGULATES INTESTINAL EPITHELIAL BARRIER IN VIVO AND PLAYS A PROTECTIVE ROLE DURING EXPERIMENTAL COLITIS

Nayden G. Naydenov[1], Alex Feygin[1], Dongdong Wang[1], John F. Kuemmerle[2], Gianni Harris[4], Mary Anne Conti[5], Robert S. Adelstein[5] and Andrei I. Ivanov[1,3,4]*

[1]Department of Human and Molecular Genetics, [2]Department of Internal Medicine, [3]Virginia Institute of Molecular Medicine, [4]VCU Massey Cancer Center, Virginia Commonwealth University, Richmond, VA 23298, [4] Department of Medicine, University of Rochester School of Medicine, Rochester, NY; [5]Laboratory of Molecular Cardiology, NHLBI, National Institutes of Health, Bethesda, Maryland 20892

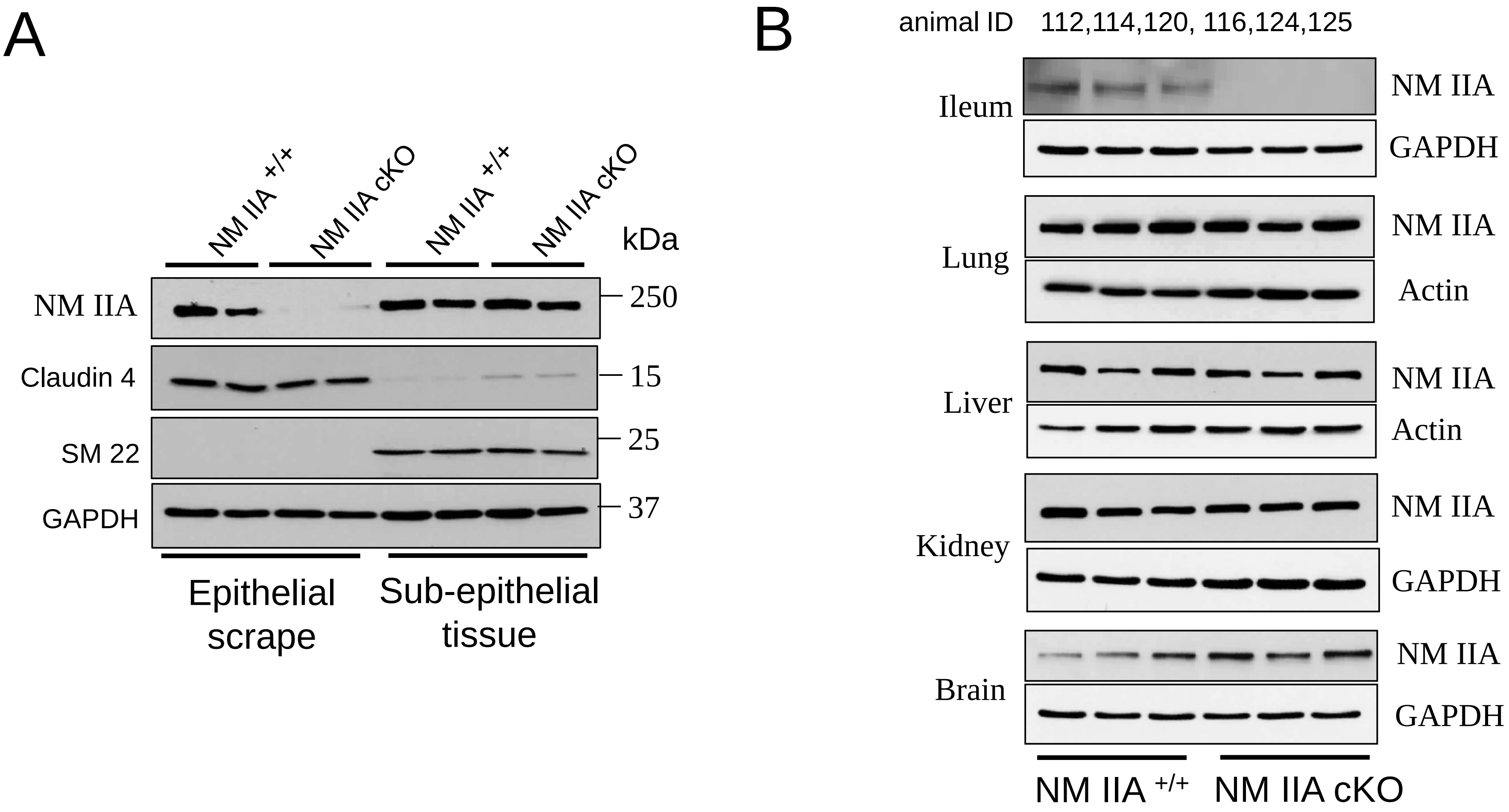

Supplementary Figure 1. NM IIA expression in different organs of control and NM IIA cKO mice. (**A**) Colonic epithelial scrapes and post-scrape submucosal/muscularis fractions of control and NM II cKO mice were subjected to immunoblotting to examine the expression of epithelial (claudin-4), mesenchymal (SM-22) markers, and NM IIA. (**B**) Immunoblotting analysis of NM IIA expression in ileal epithelium scrapes and select organs of control and NM IIA cKO mice.

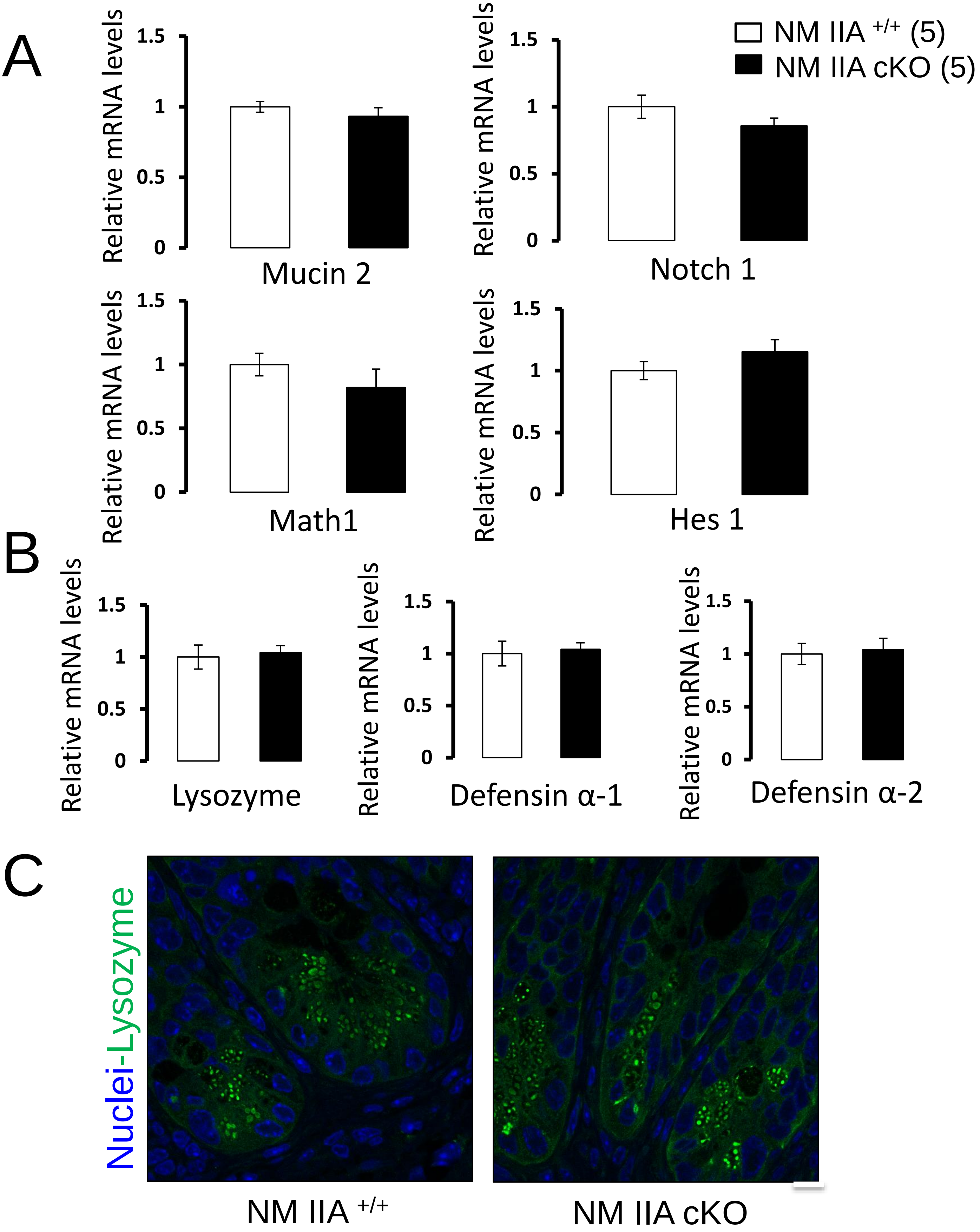

Supplementary Figure 2. The loss of NM IIA does not affect the expression of Notch-regulated genes or Paneth cell function. (A) The mRNA **e**xpression of Notch1 and Notch-regulated genes was examined using real-time quantitative

RT-PCR on whole colonic samples of unchallenged NM IIA$^{+/+}$ and NM IIA cKO animals. (**B**) The mRNA **e**xpression of different Paneth cell markers was analyzed by real-time quantitative RT-PCR on ileal samples of unchallenged NM IIA$^{+/+}$ and NM IIA cKO animals. **(C)** Paneth cell granule morphology was examined in ileal sections by Lysozyme immunolabelling (green) and confocal microscopy. Nuclear counter-staining (blue) was used to visualize the position of individual cells. The number of animals in each experimental group is shown in parentheses. Scale bar, 10 μm.

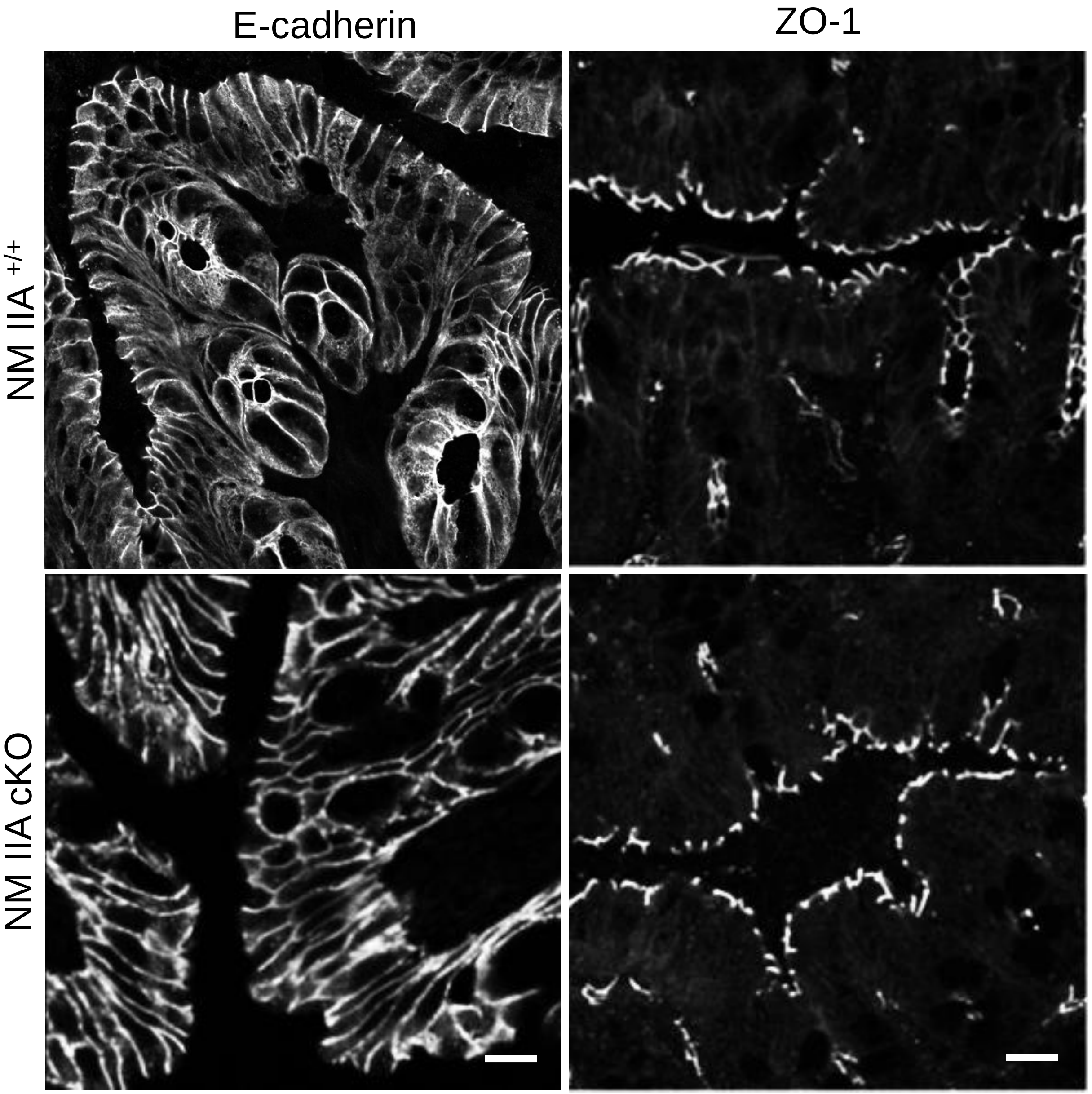

Supplementary Figure 3. Loss of intestinal epithelial NM IIA does not affect the localization of selected AJ and TJ proteins in the colonic mucosa. Colonic sections of control and NM IIA cKO animals were immunolabeled for the indicated junctional proteins and examined by confocal microscopy. Scale bar, 10 µm.

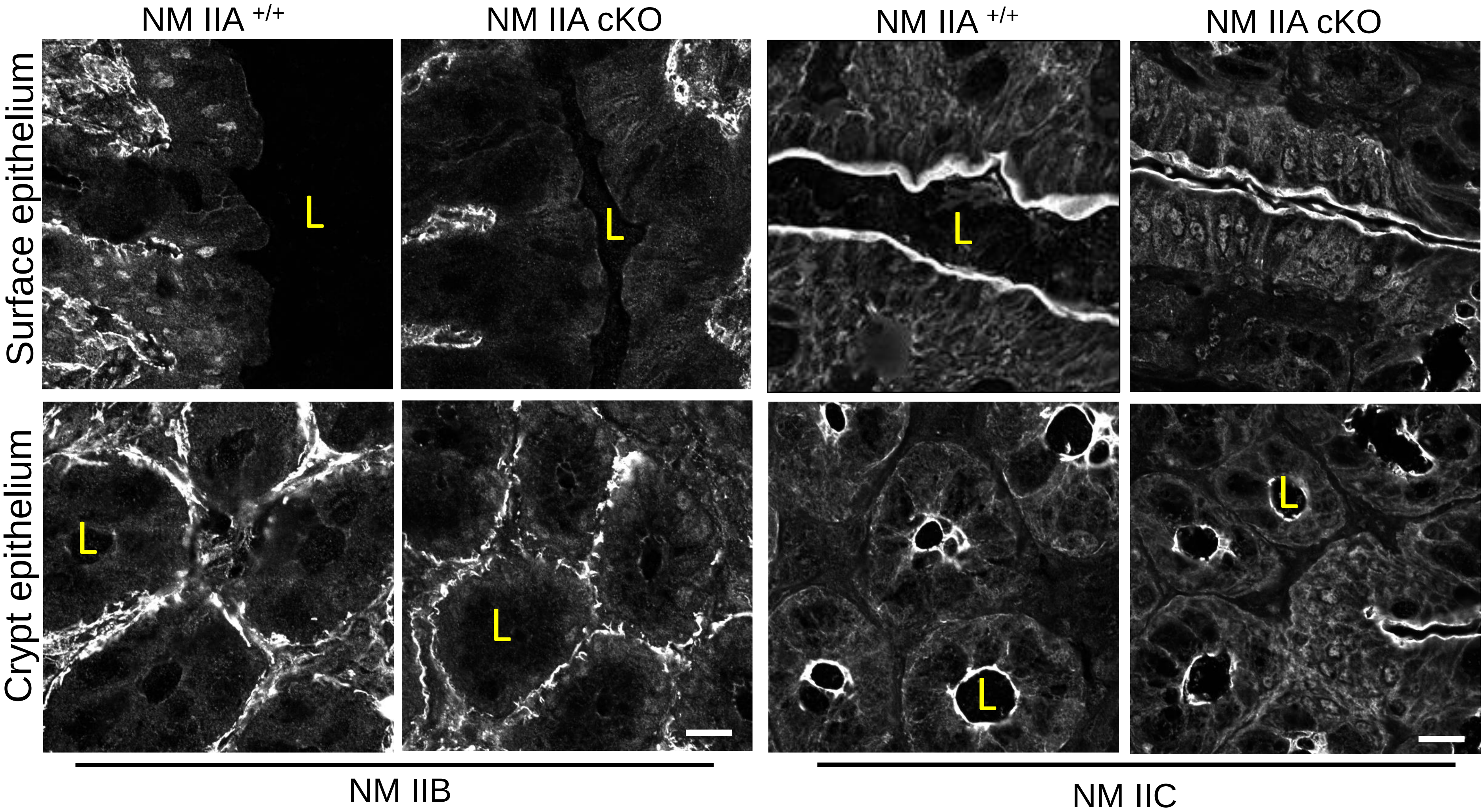

Supplementary Figure 4. Loss of NM IIA in the intestinal mucosa does not affect the localization of other NM II isoforms. Colonic sections of NM IIA+/+ and NM IIA CKO animals were immunolabeled for NM IIB and NM IIC and examined by confocal microscopy. ‘L’ indicates the gut lumen. Scale bar, 10 µm.

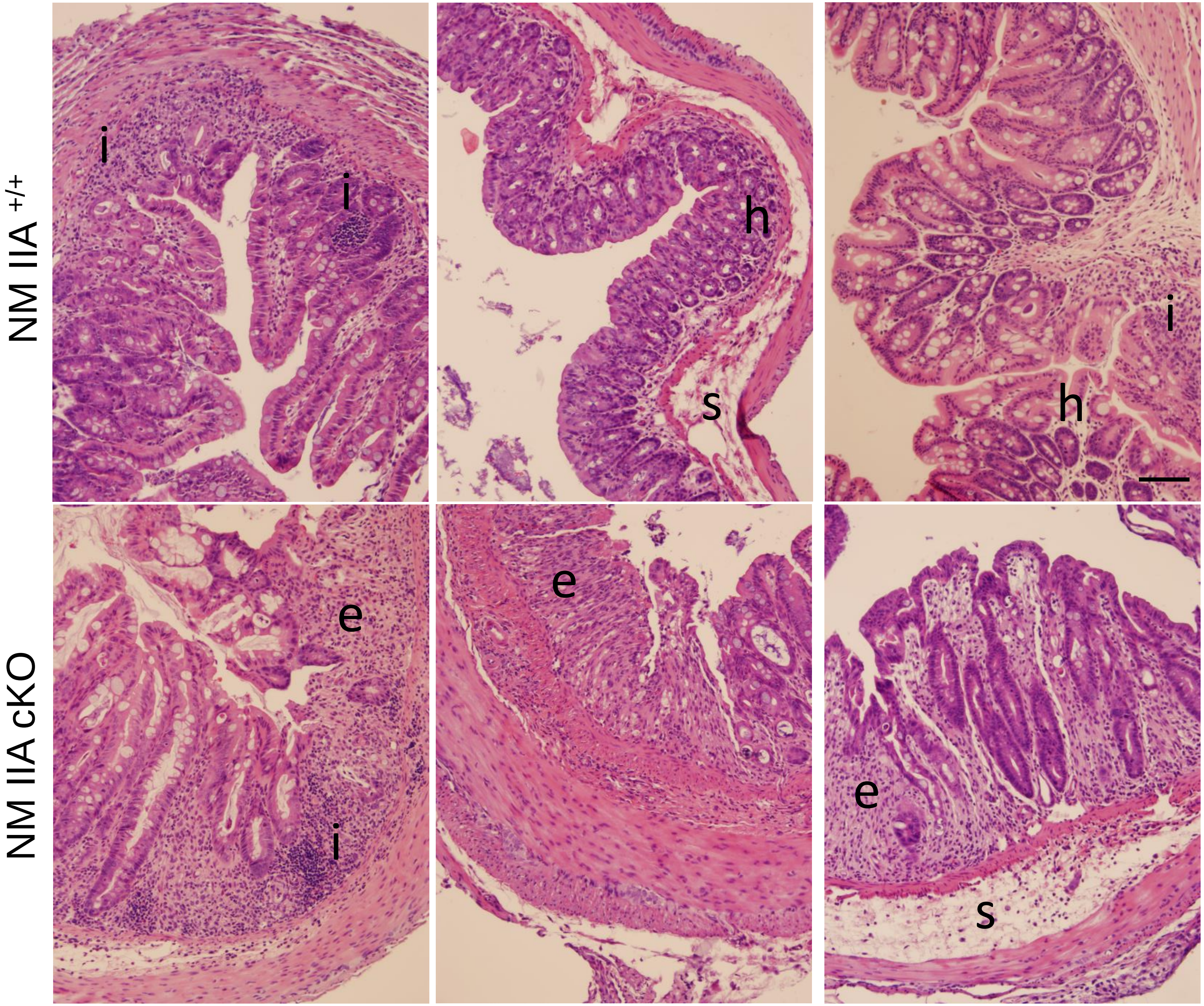

Supplementary Figure 5. Intestinal epithelial-specific knockout of NM IIA increases tissue injury during experimental colitis. This supplementary figure shows additional examples of the H&E staining of colonic

mucosal sections obtained from three different NM IIA$^{+/+}$ and three NM IIA cKO mice exposed for 7 days to 3% DSS. Changes in the tissue architecture are indicated by letters: **e**, epithelial erosion; **h**, crypt hyperplasia; **i**, leukocyte infiltration; **s**, submucosal edema. Scale bar, 50 µm.

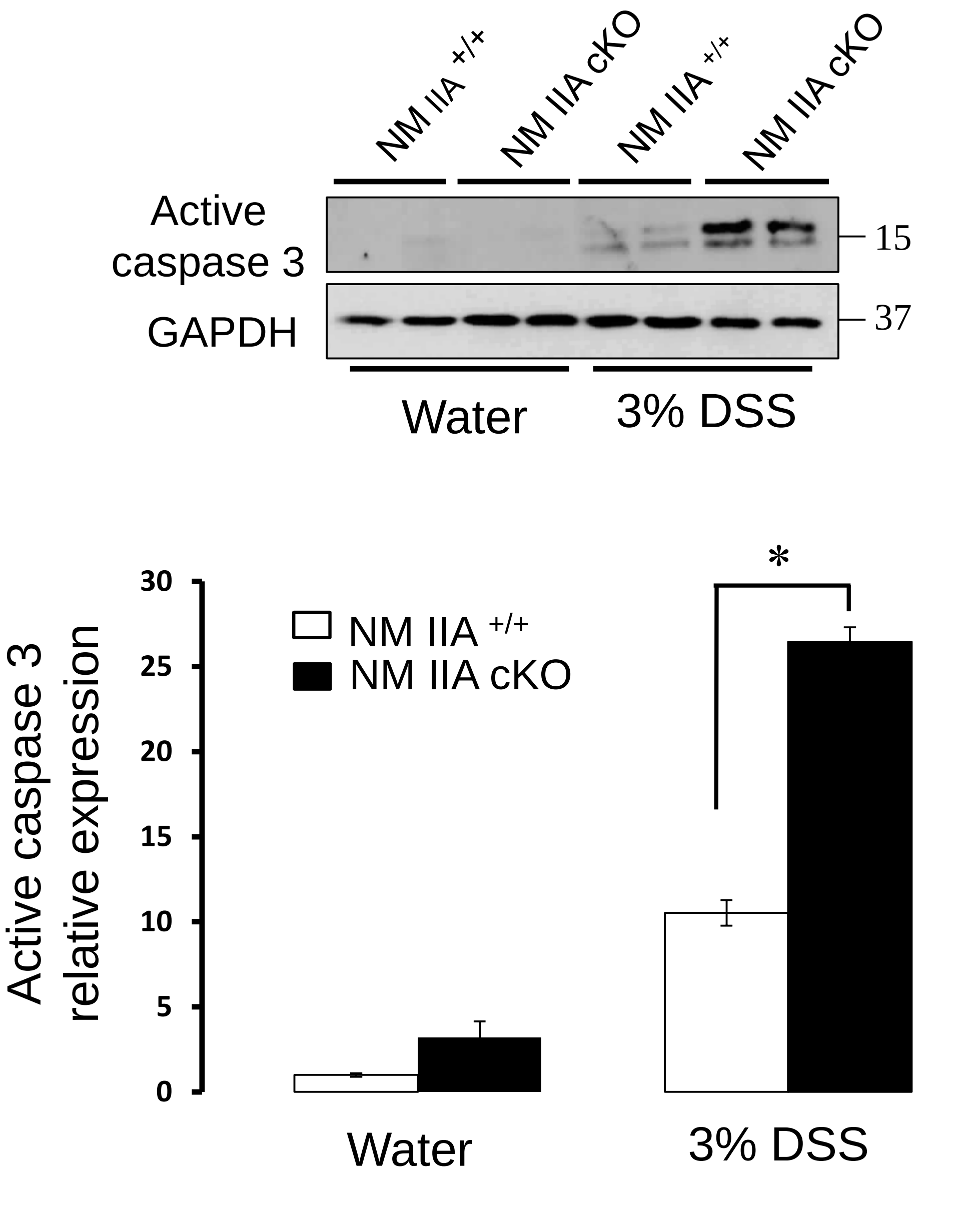

Supplemenatary Figure 6. The loss of intestinal epithelial NM IIA exacerbates epithelial cell apoptosis in the colonic mucosa of DSS-treated animals. Colonic epithelial cell scrapes were obtained from control and NM IIA cKO animals under normal conditions, and after 7 days of DSS colitis. The level of an apoptotic marker, active caspase 3, was analyzed by immunoblotting and densitometric analysis. Data is presented as mean ± SE (n = 5). *P< 0.01.

Supplemental Table S1 Primer sequences for quantitative real-time RT-PCR

Gene	GenBank No.	Direction	Primer sequence (5'-3')
GAPDH	NM008084.2	Forward	CATGTTTGTGATGGGTGTGAACCA
		Reverse	AGTGATGGCATGGACTGTGGTCAT
TNFα	BC117057.1	Forward	ACGGCATGGATCTCAAAGACAACC
		Reverse	TGAGATAGCAAATCGGCTGACGGT
IL-1β	NM_008361.3	Forward	TGGAGAGTGTGGATCCCAAGCAAT
		Reverse	TGTCCTGACCACTGTTGTTTCCCA
IL-10	NM_010548.2	Forward	GGTTGCCAAGCCTTATCGGA
		Reverse	ACCTGCTCCACTGCCTTGCT
IL-12	NM_008352.2	Forward	AGACCCTGCCCATTGAACTG
		Reverse	GAAGCTGGTGCTGTAGTTCTCATATTT
IL-17	NM_010552.3	Forward	GCAAAAGTGAGCTCCAGAAGG
		Reverse	AGCTTCCCAGATCACAGAGG
IFNγ	NM_008337.3	Forward	GCTCTGAGACAATGAACGCTAC
		Reverse	TTCTAGGCTTTCAATGACTGTGC
CCL3	NM_011337.2	Forward	CCAAGTCTTCTCAGCGCCAT
		Reverse	GAATCTTCCGGCTGTAGGAGAAG
CXCL5	NM_009141.3	Forward	CTCAGTCATAGCCGCAACAGC
		Reverse	CGCTTCTTTCCACTGCGAGC
COX2	NM_011198.4	Forward	TGAGTACCGCAAACGGTTCTC
		Reverse	TGCAGCCATTTCTTTCTCCT
KC	NM_008176.3	Forward	CTTGAAGGTGTTGCCCTCAG

		Reverse	TGGGGACACCTTTTAGCATC
MIP-2	NM_009140.2	Forward	GGCAAGGCTAACTGACCTGGAAAGG
		Reverse	ACAGCGAGGCACATCAGGTACGA
Lysozyme	NM_013590.4	Forward	GTCACTGCCCAGGCCAAGGT
		Reverse	CGGTGCTTCGGTCTCCACGG
Defa-rs1	NM_007844.2	Forward	TGCCCTCGTTCTGCTGGCCT
		Reverse	AGCAGAGCCTTCTGTGCCTCCA
Defa-2	NM_001195634.2	Forward	TGGCCTTCCAGGTCCAGGCT
		Reverse	CCTGGTCCTCCTCCCCTGGC
Tgfβ1	NM_011577.2	Forward	ACCATGCCAACTTCTGTCTG
		Reverse	CGGGTTGTGTTGGTTGTAGA
mMUC2	NC_000073.6	Forward	GGGAGGGTGGAAGTGGCATTGT
		Reverse	TGCTG GGGTTTTTGTGAATCTC
mMath1	NC_000072.6	Forward	AGCTGTCCAAATATGAGACCCTACA
		Reverse	GACATTGGGAGTCTGCAGCAA
mHES1	NC_000082.6	Forward	CAGCTCCGGGAAAGCAAGCCC
		Reverse	GCCACCTTTCTCTGAGTCACCGC
NOTCH1	NC_000068.7	Forward	CATGGGCGCACAGGTCTGCT
		Reverse	AGGGGCAGGTGCAGATGGCT
